# Fast, Ungapped Reads Mapping Using Squid

**DOI:** 10.3390/ijerph19095442

**Published:** 2022-04-29

**Authors:** Christopher Riccardi, Gabriel Innocenti, Marco Fondi, Giovanni Bacci

**Affiliations:** 1 Department of Biology, University of Florence, Via Madonna del Piano 6, 50019 Sesto F.no, Florence, Italy; christopher.riccardi@unifi.it (C.R.); gabriel.innocenti@stud.unifi.it (G.I.); 2 Institute for Molecular Bacteriology, TWINCORE Centre for Experimental and Clinical Infection Research, A Joint Venture between the Hannover Medical School (MHH) and the Helmholtz Centre for Infection Research (HZI), Hannover, Germany

**Keywords:** dynamic programming, rna-seq, mapping, quality check

## Abstract

Advances in Next Generation Sequencing technologies allow us to inspect and unlock the genome to a level of detail that was unimaginable only a few decades ago. Omics-based studies are casting a light on the patterns and determinants of disease conditions in populations, as well as on the influence of microbial communities on human health, just to name a few. Through increasing volumes of sequencing information, for example, it is possible to compare genomic features and analyze the modulation of the transcriptome under different environmental stimuli. Although protocols for NGS preparation are intended to leave little to no space for contamination of any kind, a noticeable fraction of sequencing reads still may not uniquely represent what was intended to be sequenced in the first place. If a natural consequence of a sequencing sample is to assess the presence of features of interest by mapping the obtained reads to a genome of reference, sometimes it is useful to determine the fraction of those that do not map, or that map discordantly, and store this information to a new file for subsequent analyses. Here we propose a new mapper, which we called Squid, that among other accessory functionalities finds and returns sequencing reads that match or do not match to a reference sequence database in any orientation. We encourage the use of Squid prior to any quantification pipeline to assess, for instance, the presence of contaminants, especially in RNA-Seq experiments.

## 1. Introduction

The natural course for short reads produced by Next Generation Sequencers (for example Illumina) is for them to be processed according to base quality and adapters content, then assembled or mapped, depending on the application (for example, transcripts quantification or genome sequencing). When assembly is performed de novo, algorithms are applied with the end goal of producing long *contiguous* pieces of sequence (contigs) from the short reads [1]. Other software allows for the reconstruction of a genome or transcriptome using a reference-based approach to correct misassemblies or for variant detection [2,3]. Instead, when mapped or aligned, the reads are compared to a DNA sequence, usually a genome or an exome, for features counts or transcript quantification. Throughout these applications, sequencing contamination is an undermining factor that, if not taken into account, can severely hamper downstream analyses. Although most NGS platforms handle reagents automatically, the modern sequencing process is still prone to human error or contamination. This can derive from previously manipulated samples that may leave traces during the amplification process, as in the case of DNA contamination in an RNA-Seq protocol [4]. The sources of these include machine or equipment contamination, intrinsic preparation/sequencing or computational errors. Recent studies [4] have also reported transcripts deriving from the same tissue that were heavily contaminated with other (non-self) samples deriving from the Genotype-Tissue Expression (GTEx) project [5]. It is also interesting to see how publicly available software has undergone a profound change over the years to better exploit sequencing data. Today, graph-based assemblers are tailored in such a way that they tend to handle a larger number of short reads with proper optimization when compared to the long reads produced by Sanger sequencing. Computational tools such as fastp [6,7] not only allow us to filter reads by per-base quality, for example the probability of incorrect base call, but they can also manipulate FASTQ files to remove reads that are duplicated or that are shorter than a user-defined size, other than reporting loads of useful information. Considerable effort has been spent on creating read aligners that are able to report mappings in Sequence Alignment Map (SAM/BAM) formats [8], such as Bowtie2 [9], STAR [10] or Magic-BLAST [11], from which it is possible to extrapolate intrinsic information relative to reads that mapped discordantly, or that did not map at all. Within the context of this latter application, to the best of our knowledge, no peer reviewed standalone tool exists that allows a fast, straightforward, and customized handling of sequencing reads with respect to their mapping orientation. Retrieving this information (read orientation relative to the reference sequence for single ended sequencing, and relative to each other in the case of paired ended reads) from the bitwise flags in the most used SAM/BAM file formats is an elementary procedure for a bioinformatician but can get quite laborious for non-specialized researchers. Moreover, combining the mapping, filtering, and sorting of sequencing reads against coding sequences or relatively small genomes (bacterial and some yeasts for example) can serve as a quick assessment tool even for wet lab scientists who are in charge of the preliminary analyses. It is for this reason that we propose a novel multi-platform, dependencies free software called Squid for fast ungapped mapping of NGS reads in the FASTQ format. Squid software can split a sample from either a single end or paired end sequencing into a matching or non-matching new file in the FASTQ format. For matching sequencing reads, it is also possible to write the mapping positions to a Browser Extensible Data (BED) format, which makes it easy to manipulate and read coordinates during post-processing stages of the analyses. Moreover, it does not require the user to write and load the database index in separate steps, as it performs database indexing and search all at once. Noticeable speed is gained due to the fact that it can report a matching read (or pair of reads) as soon as they are found in the database without mapping them to multiple locations, as it would happen with most read mapping tools that generate a SAM/BAM file. The current implementation offers an ungapped mismatch alignment and therefore, in this sense, Squid should not be considered as an aligner but, rather, as a mapper that can split reads according to their alignment against a given reference database. Squid was designed to be highly intuitive, and to be able to handle sequencing reads that have been already trimmed or filtered in any way, without the need of a pedantic user input. Moreover, it offers a number of combinatorial options that make the output fully customizable while maintaining an exceptional speed.

## 2. Materials and Methods

### 2.1. General Design

Downloading, compiling, and running Squid is extremely easy. All of the tool’s optional parameters and functionalities are explained at the GitHub repository [12] https://github.com/combogenomics/Squid (accessed on 20 April 2022). In order to run Squid, the user will simply need a sequence in FASTA format to be used as a reference, and a sample in either single or paired end (FASTQ). Squid supports reference and input reads either in an uncompressed or compressed (gzip) format, through the inclusion of the zlib C Application Programming Interface (API). The user can adjust the preferred orientation to return sequencing reads that match the reference in the forward direction, for example the given input, or the reverse complement. When in paired end mode, it is possible not only to take the sample library protocol into account (stranded or not stranded), but to return a read pair when the two are oriented towards or opposite to each other. In an RNA-Seq experiment that adopted a forward stranded protocol, for example, using the library option “-l ISR” and providing a multi-FASTA with the coding sequences (CDS), allows us to retrieve reads that match the reverse complement of a CDS, thus potentially representing a source of contamination or error (see “Read orientation modes” paragraph). When a sequencing read is found while scanning the reference database, depending on the selected option, Squid will either write or retain the read that returned a positive match. It achieves this through a *hit-and-extend* approach using seeds of tunable length and extending them during a subsequent ungapped mismatch search. Database seeds, or *kmers*, are stored in what we refer to as a “pseudo-hash table” (a sorted array of numerical hashes which is accessed using a binary search). The sequencing read is also broken down into several *kmers* using a user-defined sliding window. 

### 2.2. Implementation

Squid software is a command line tool, and it is written in the C programming language. Much of the speed comes from the fact that Squid utilizes a novel pseudo-hash function for multi-seed binary search in a *hit-and-extend* approach. The pseudo hash function is used during database indexing and during multi-seed search. Squid enables to keep or discard sequencing reads that match a given database reference in any orientation, which makes it suitable for assessing contaminants or spurious products that were not intended to be sequenced. Squid can produce BED and BED paired end (BEDPE) file formats, as well as FASTQ. This, together with a complete set of tunable parameters, allows to easily assess problems that could have derived from library preparation or upstream procedures, that are sometimes challenging to detect.

### 2.3. The Pseudo-Hash Function

If a hash function maps data of arbitrary size into fixed-size values, then our function is a pseudo-hash, as it shrinks words of a *fixed* size from an *A*, *C*, *G* and *T* alphabet into a numerical value.

The pseudo-hash function reads the nucleotide reference sequence by stretches of *k*-length and assigns an *unsigned int* to each stretch, as such:

(1) $$y=\overset{i=k-1}{\underset{i=0}{\sum }}N_{i}\cdot 4^{\left(k-1\right)-i},\;\;\;\;N\;\in \left\{A=0;C=1;G=2;T=3\right\}$$

where *y* is the produced pseudo-hash and *k* the kmer length. Please note that gaps and *Ns* are not taken into account by the hash function, which means that they cannot represent a hit during the pseudo-hash matching phase, but they are simply treated as mismatches during the extension phase. For a faster computation, the exponents are pre-calculated, leaving the user with a choice between a kmer (*k*) of 9, 11, 13 and 15. The hashes are then sorted using a recursive merge sort algorithm, which is one of the fastest having an average and worst-case performance of O (*n* log *n*). The pseudo-hash table is sorted so that elements accessing can be as little time consuming as possible using a logarithmic search which instead, in the worst case, runs as O (log *n*). Efficiency is also gained by the fact that hashes are integral types, the difference of which is very straightforward to compute.

### 2.4. Seed Ungapped Extension

Each sequencing read is seeded into a pre-determined number of fragments (*r*), that is a function of the read length (*l*), the fragment length (*k*) and the step size (*s*):

(2) $$r=\frac{1+l-k}{s}$$

Whilst the read length is allowed to change, the remaining parameters are provided through the command line, and can be tuned to achieve a higher degree of accuracy. Given a fragment, there are three possible outcomes at implementation level. The first scenario is the one in which the (native C) binary search function does not return a match in the hash table; in such case the next-in-queue fragment of size *k* at a distance *s* from the previous, is searched iteratively. In the second scenario, the binary search returns a match, and the sequencing read is aligned and compared to the reference sequence. This is possible because the pseudo-hash table stores the position at which every fragment was found, allowing for the ungapped extension to proceed. There is the possibility that the read being compared does not align properly to the reference sequence in correspondence of which the seed was found. In this case, the next collision in the pseudo-hash table is probed until all colliding sequences having the same seed are tested. If no match was produced, Squid jumps back to the seeding stage, sliding to the next fragment at another distance *s*, and repeating the entire search a certain number of times, up to *r*. The current implementation does not allow to change the *r* value, and it is set for the most exhaustive search possible. The third scenario is the one in which a fragment is found, and the read aligns well (respects the maximum allowed mismatches) to the rest of the database reference sequence. The type of search we implemented allows for mismatches to occur with same probability at any position, regardless of the read quality. The user can decide the maximum percentage of mismatches that are allowed to occur, and the search function breaks early out of the loop if that threshold is achieved iteratively. The number of misaligned bases to use as a reference is computed at every cycle to ensure consistency even with reads that were trimmed and that don’t have consistent length.

### 2.5. Reads Orientation Modes

**Figure 1:**
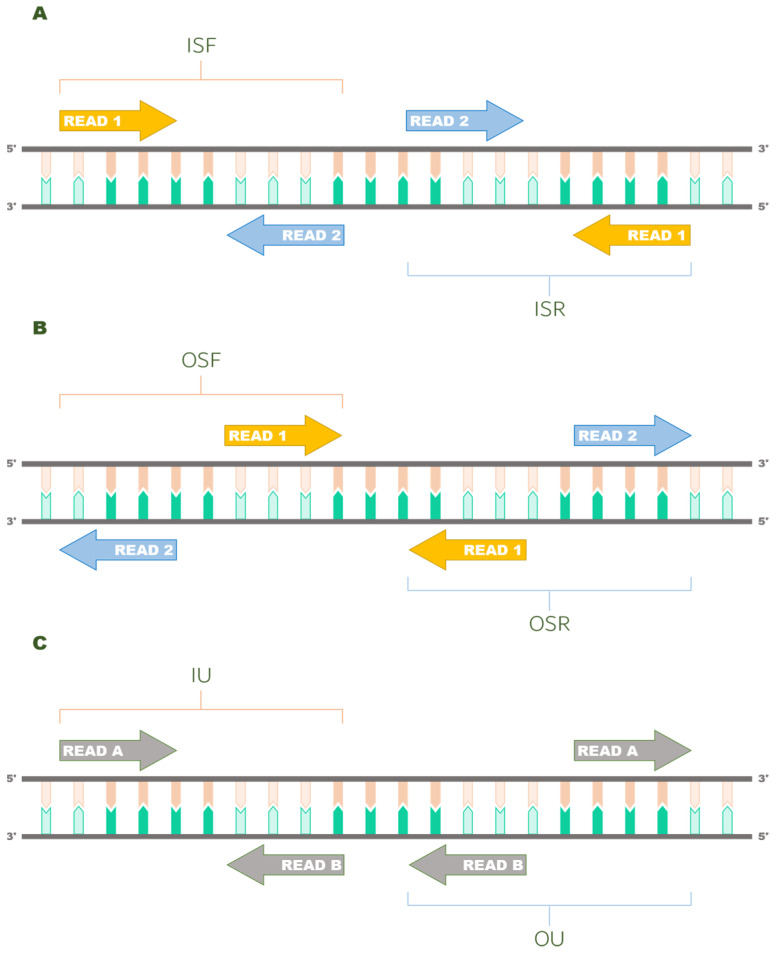
**Read orientation modes handled by Squid**. Library types ISF, ISR (**A**) and IU (**C**) model fragments in which the sequencing reads are oriented towards each other. Read A and B represent any R1–R2 pair, as long as mutual exclusivity and orientation are conserved. Library types OSF, OSR (**B**) and OU (**C**) instruct Squid of the opposite case, in which the sequencing reads do not face one another. A fragment is never modelled in a matching library protocol (both reads mapping to the same strand) in the current implementation.

Typically, read libraries can be prepared with a stranded or not stranded protocol. To ensure maximum compatibility with existing libraries, we have implemented a set of nine library format strings that the user can adopt, using the approach implemented in Salmon [13] as a blueprint. The first letter of the format strings applies only if the library is paired ended, and it specifies the relative orientation of the sequencing reads. When “I” is specified, the fragment is modelled through an inward conformation in which the reads are oriented towards each other (Figure 1A). On the contrary when “O” is specified, the resulting fragment is modelled in such a way that the sequencing reads are oriented opposite to one another (Figure 1B). The second letter indicates whether the sample library comes from a stranded (“S”) or a not stranded (“U”) protocol. This part of the library format string also applies to single ended reads, and when U is specified, the read is scanned in its forward and reverse orientation to the reference. The third letter is a rule for forcing to scan the forward, reverse or both strands for every read type, according to the chosen sample library preparation.

### 2.6. The Exhaustiveness Parameter

When the researcher that uses Squid is interested in splitting the sequencing reads according to a database reference match, it is not necessary to explore other mapping locations other than the first with a sufficient score. If, however, quantification is at play, then it is reasonable to report the best alignment among a number that are of comparable goodness. We implemented the possibility to explore more than one fragment mapping position when in paired end mode, through the *-e* flag, which stands for *exhaustiveness*. This option allows to reiterate the search up to *int* times, looking for a match with the best cumulative score, among the explored solutions. This could mean that Squid will report a better scoring match within the same sequence (less likely) or in another sequence that was present in the multi-FASTA database. Increasing the exhaustiveness allows for a better representation of sequences in the database that share a significant similarity, at the natural expense of performance.

### 2.7. Simulated Data Preparation

In order to benchmark our tool, we simulated one RNA sequencing experiment with differential transcript expression and biological replicates using the Polyester R package (version 4.0.3). The first 100 transcripts from human chromosome 22 (hg19, for a total of 2,733,262 nucleotides) were collected and raw RNA-Seq reads measuring 100 bases in length were simulated. The function *simulate_experiment* was run with a set of 100 random fold changes, applying a uniformly distributed error rate of 0.5%, with increasing library size factors, to generate 10 paired ended samples of different size.

### 2.8. Real Data Collection

We downloaded *Escherichia coli* K12 substr. MG1655’s total RNA sequencing run with identifier SRR12518293 (paired ends, 42.1 M spots, 12.6 Gbp) from the Sequence Read Archive (published 4 December 2020, last accessed 4 February 2022), CDS and rRNA sequences from the genomic assembly with identifier ASM584v2, submitted by the University of Wisconsin on 26 September 2013, and last accessed on 4 February 2022.

### 2.9. Benchmarking Software Parameters

When testing for performance, Bowtie2 version 2.2.9 was run in—*very-sensitive* mode. Both programs were run on single AMD Opteron 6380 processor (2.5 GHz) on the ten simulated replicates, setting a *kmer* size to 9 and a sliding window of 1 nucleotide. The “-k” parameter in bowtie2 was set to 1 to only report one alignment per read, the same was set for Squid using *-e 0*. Salmon version 1.6.0 was run in selective alignment mode through the *–validateMappings* flag during the accuracy benchmarking stage to ensure maximum sensitivity. *Kmer* length was set to 9 nucleotides to ensure consistency.

## 3. Results

### 3.1. Performance Benchmarking

Bowtie2 performs a gapped alignment and a true indexing of the input database reference sequence with a relatively low memory footprint. Its purpose serves a wide range of applications, and it is debatable whether the two programs could be functionally comparable. Nonetheless, we measured the execution time of both programs on a simulated dataset based on human chromosome 22 transcripts (see Materials and Methods). This type of non-competitive benchmark allows to estimate Squid’s performance when it comes to dealing with high throughput NGS data (Table 1). In all cases, Squid outperformed Bowtie2 with a speedup ranging from 5.45 to 7.26 folds. Squid took roughly 2 minutes to work on Sample S_10, which counted more than 2 million paired reads, whereas Bowtie2 took approximately 12 minutes. When taking into account the percentage of mapped reads, both programs mapped 100% of the sequencing reads. We would point out, however, that the current implementation does not perform a gapped alignment and Bowtie2 is superior in terms of accuracy when it comes to spotting indels. Nonetheless, when looking for ungapped alignments with some degree of mismatches, Squid is superior in terms of performance.

### 3.2. Accuracy Benchmarking

**Figure 2:**
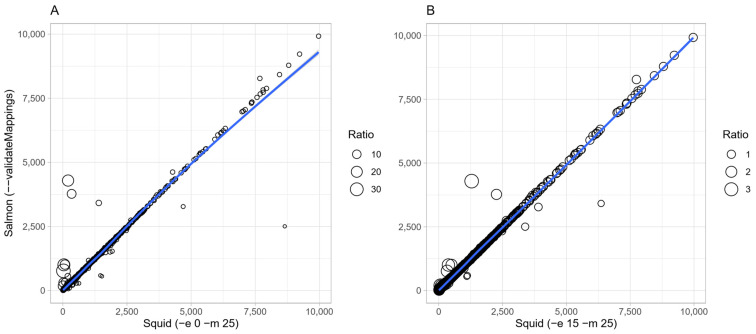
**Scatterplot of transcripts quantification**. Each circle represents the raw counts of a gene in Salmon (*y* axis) and Squid (*x* axis). Squid was run using exhaustiveness 0 (**A**) and 15 (**B**), respectively. Per-gene ratio was calculated dividing Salmon’s raw counts by Squid’s raw counts (extracted from the BEDPE output file). Note how the ratio scale is different by an order of magnitude between (**A**) and (**B**), indicating that mapping accuracy is affected when no additional cycles are performed. Coefficients of determination were 0.97 and 0.99 in (**A**) and (**B**), respectively. The regression line was calculated using the generalized additive model (GAM) through the R package ggplot2.

We sought to evaluate our software’s accuracy by downloading a real dataset and testing how well Squid distinguished reads that map to ribosomal RNAs or coding sequences in an RNA sequencing experiment. The library construction protocol for sample SRR12518293 included a rRNA depletion step, but we suspected a significant presence of ribosomal cDNA sequences. We first used fastp for quality check and trimming of the reads (starting set). We then downloaded the transcriptome file from the NCBI and built a non-redundant Salmon index providing the entire genomic sequence as a decoy. After these preparation steps, we launched Salmon on the starting set of reads in a selective alignment and quantified the number of transcripts assigned to each feature. The recorded mapping rate was 98.22% and the percentage of reads, over the total mapped, aligned to rRNAs was 99.93%, whereas those aligned to CDS were only 0.06%. Next, we used Squid with its–*diff* option to dump reads from the starting set that do not match the rRNA reference sequence file. The latter was given directly to the command line since no index build is required to run Squid; the search was performed using 32 cores. Squid produced 4,417,176 (10.61%) reads flagged as not matching the rRNA database (the difference set, rRNA-free sequencing reads). Finally, Salmon was used to assess the presence of the residual ribosomal RNAs on the difference set. Salmon detected 2.47% of the reads still mapping to rRNA sequences, but 97.53% of them mapping to CDS. According to this simple benchmark, Squid could remove almost the entire set of RNA contamination-derived reads. We then intended to measure how well Squid could map the starting set of reads to the non-ribosomal gene transcripts used in the previous analysis. For the purpose of this latter assessment, we utilized the previously obtained Salmon quantification file, deprived of the rRNAs sequences; the resulting raw counts served as the oracle dataset for mapping correctness evaluation. Squid was run several times, gradually increasing the exhaustiveness parameter from an initial value of 0 (first match is good to go) up to 50 evaluations per sequencing read pair, keeping the maximum allowed percentage of mismatches at 25%. Increasing the exhaustiveness allowed for a more thorough search for the best match among possible mappings (Figure 2).

With no additional cycles, Squid produced stronger outliers than when 15 were allowed (*-e* parameter set to 0 and 15, respectively), even though there is still a nearly perfect positive correlation between each run and the oracle dataset. It is worth mentioning that an R^2^ value of 0.99 was already achieved when the exhaustiveness was set to at least 5 (data not shown).

### 3.3. Study of the Outliers

The underlying reason of the shift in ratio that is observable in Figure 2 is biological, for the most part. We manually picked the top 5 outliers that showed a ratio greater than 2 or less than 0.5 and performed a functional search on the KEGG database [14]. Moreover, we evaluated whether there was any sequence similarity between and among these genes and the rest of the non-redundant genome, making use of the Average Nucleotide Identity (ANI) values, calculated using fastANI [15]. Each of the 5 genes showed >98% similarity to at least one other gene with which it shares the same KEGG pathway, namely gene pairs: *insB4*-*insB1* (98.3%), *insI2*-*insI1* (99.74%), *tfaQ*-*tfaR* (99.83%), *rhsA*-*rhsB* (99.4%) and *insH6*-*insH1* (94.5%). In order to understand whether the resizing of the outliers from *-e* 0 to *-e* 15 can be attributed to a more precise mapping of reads to similar sequences, we measured the gene pairs’ correlation at increasing levels of exhaustiveness. Using Pearson’s correlation coefficient for every pair of raw count vectors we could observe a strong anticorrelation (coefficient of −1 in 100% of cases, data not shown), which further supports the hypothesis that Squid was able to find a better match between similar sequences by iterating through multiple possible solutions.

### 3.4. Concluding Remarks

The current implementation of Squid is designed to rapidly distinguish between mapping and non-mapping reads, but we are already working on a gapped version that would provide a higher resolution of Next Generation Sequencing data. We have tested our software on gene sequences deriving from a human chromosome as well as on bacterial coding sequences. The pseudo-hash table that we have implemented ensures great speed on reference sequences of such size and composition, making Squid ideal for fast ungapped mapping of reads on reference sequences by the order of magnitude of Bacteria or eukaryotic CDS. The hereby presented partitioning and filtering of sequencing data is currently possible using several programs in a pipeline, and our software tries to do all at once through some algorithmic simplifications which are traded off for increased speed. Nonetheless, it has proved to be up to par with some of the most advanced resources for sequencing data analysis, making it a valid alternative for quality assessment tasks.

Finally, we would point out that besides the one benchmarked here (Bowtie2), many alternative aligners exist, including, graph based aligners (for example, HISAT2 [16]), suffix array based transcriptome aligners (for example., STAR [10]), suffix tree/suffix array based genome aligners (for example, MUMmer [17]) and RNA-seq aligners implemented to correctly map reads that present variable length indels on eukaryotic transcriptome (for exampl, TopHat2 [18]). We believe that Squid represents a valuable alternative to these complex and very specific aligners as it relies on a simple, flexible, and fast implementation to help researchers in routinary and basic NGS data analysis such as contaminant detection in reads files.

## Figures and Tables

**Table 1:** **Squid’s performance compared to Bowtie2**. The average reported speedup is 6.24; both programs were set to operate with a *kmer* length of 9 and step size of 1 nucleotide. Bowtie2 was set to ignore quality scores since Squid does not consider them in the current implementation. Moreover, a reporting value of *k =* 1 was selected in Bowtie2 to only report the first alignment found, and not look for multiple alignments. Squid library string “−l IU” ensured that all of the inward orientations of the read pairs were to be detected.

	Reads	Time (s)		Percentage Mapped		Speedup
Sample		Squid	Bowtie2	Squid	Bowtie2	
S_01	294,551	18.14	98.91	100	100	5.45
S_02	428,090	25.00	149.04	100	100	5.96
S_03	433,494	24.61	156.52	100	100	6.36
S_04	477,233	24.16	163.3	100	100	6.75
S_05	568,939	33.70	216.28	100	100	6.41
S_06	822,335	50.38	280.41	100	100	5.56
S_07	1,056,705	52.42	365.25	100	100	6.96
S_08	1,611,954	75.84	550.62	100	99.99	7.26
S_09	1,933,648	97.18	647.1	100	100	6.65
S_10	2,261,861	111.65	755.5	100	100	6.76

## Data Availability

Squid supports multithreading and runs on Mac OSX and Linux kernel. Open-source code and documentation are available at [12] https://github.com/combogenomics/Squid (accessed on 20 April 2022).
